# Comparison of PD-L1 expression in squamous cell cancer of unknown primary and oropharyngeal squamous cell carcinoma

**DOI:** 10.1007/s00405-022-07775-z

**Published:** 2022-12-28

**Authors:** Benedikt Schmidl, Kim-Aylin Voßenkämper, Leonhard Stark, Melanie Boxberg, Fabian Stögbauer, Katharina Feigl, Christof Winter, Anja Pickhard, Barbara Wollenberg, Markus Wirth

**Affiliations:** 1 Department of Otolaryngology Head and Neck Surgery, Technical University Munich, Munich, Germany; 2 Institute of Pathology, Technical University Munich, Munich, Germany; 3 Pathologie Muenchen Nord, Munich, Germany; 4 Institute of Clinical Chemistry and Pathobiochemistry, Technical University Munich, Munich, Germany

**Keywords:** HNSCC, OPSCC, SCCUP, PD-L1, Immunotherapy, HPV, Tumor microenvironment

## Abstract

**Purpose:**

The tumorigenesis of squamous cell cancer of unknown primary (SCCUP) in the head and neck area has not been decoded so far, while poor survival rates and limited therapeutic options pose a serious challenge. The aim of this project was to investigate immunological characteristics of SCCUPs and compare them to oropharyngeal squamous cell carcinoma (OPSCC).

**Methods:**

PD-L1 expression (TC) was examined by immunohistochemistry in 50 lymph node metastases of SCCUP and 47 primaries of OPSCC. CD3 + and CD8 + lymphocytic infiltration was measured in 5 high power fields. Expression of p16 and HPV ISH were assessed.

**Results:**

SCCUP demonstrated a significantly higher expression of PD-L1 than OPSCC. In p16-negative SCCUPs PD-L1 proved to be an independent prognostic factor to prioritize high-risk patients.

**Conclusions:**

Immunologic differences between SCCUP and OPSCC were detected. A higher PD-L1 expression in SCCUP could potentially facilitate further evaluation of checkpoint inhibitor therapy.

**Supplementary Information:**

The online version contains supplementary material available at 10.1007/s00405-022-07775-z.

## Introduction

The tumorigenesis of squamous cell cancer of unknown primary (SCCUP) in the head and neck area has not been decoded so far, while poor survival rates and limited therapeutic options pose a serious challenge [1, 2]. The aim of this project was to investigate immunological characteristics of SCCUPs and compare them to oropharyngeal squamous cell carcinoma (OPSCC); (2) Methods: PD-L1 expression (TC) was examined by immunohistochemistry in 50 lymph node metastases of SCCUP and 47 primaries of OPSCC. CD3 + and CD8 + lymphocytic infiltration was measured in 5 high power fields. Expression of p16 and HPV ISH were assessed; (3) Results: SCCUP demonstrated a significantly higher expression of PD-L1 than OPSCC. In p16-negative SCCUPs PD-L1 proved to be an independent prognostic factor to prioritize high-risk patients; (4) Conclusions: immunologic differences between SCCUP and OPSCC were detected. A higher PD-L1 expression in SCCUP could potentially facilitate further evaluation of checkpoint inhibitor therapy. The incidence of cancer worldwide is more than 24.5 million cases, resulting in 9.6 million deaths per year. This number increased by more than 30% between 2007 and 2017 [3]. Approximately 2–4% of all cancers are defined as cancer of unknown primary (CUP). Distinct clinical features include early metastatic dissemination in an atypical pattern, poor response to conventional chemotherapy and aggressive progression [3]. In the head and neck region up to 9% of malignancies are considered as CUP, mainly with squamous cell appearance (53–77%). Even though using an extensive diagnostic workup, the corresponding primary tumor of a lymph node metastasis cannot be found. Presumably due to better imaging, the incidence of CUP is decreasing, but the difficulty of diagnosis and treatment still poses a big challenge [4, 5]. Thinking of CUP as the metastasis of a primary tumor, identification of the primary tumor is the most important aspect of improving survival and quality of life in patients. The theory of a concealed primary is supported by reports, that a majority of primaries could be found in autopsy studies [6]. The hypothesis of CUP as a metastasis of an (still) occluded primary is also supported by the fact, that around 15% of CUPs resemble a cancer of known origin [4, 7].

Similar to HNSCC, a large proportion of CUP cases present themselves for the first time in the form of cervical lymph node metastasis [8]. Due to the lymphatic drainage of the pharynx, the most probable location of a presumed primary of SCCUP in the neck levels II–IV is the pharynx [9]. Most cases initially present with a neck mass or other metastasis-related symptom, such as weight loss, malaise or fatigue [10]. As these symptoms do not point to a specific location, the next steps are thorough clinical and radiological diagnostics and histopathological examination of the lymph node tissue [7]. Epstein–Barr virus-encoded RNA (EBER) and p16, a surrogate marker for HPV, are assessed [11]. p16 is important, as an infection with HPV is common in squamous cell carcinomas of the oropharynx [12]. EBER on the other hand is linked with carcinogenesis of nasopharyngeal carcinoma, another potential site of origin [13]. Since oropharyngeal squamous cell carcinoma (OPSCC) are the most common HNSCC [14], SCCUP were compared to OPSCC in this study. Diagnostic imaging then allows the evaluation of the extent of the disease. Treatment of cervical metastasis is still controversial, since randomized trials are not yet available [15]. Unilateral neck dissection or radiation is often performed as initial treatment. In addition, a tonsillectomy and base of tongue mucosectomy are also viable options [11, 12]. In the palliative setting, the response rates to platin-based chemotherapy are limited and alternative therapies are needed to improve the survival of patients [15]. The heterogeneous molecular character of CUPs has defied a “one size fits all” solution in the past [16] and emphasized therapy based on the individual molecular landscape.

In HNSCC recent findings highlighting the importance of the tumor microenvironment and its interactions with the tumor cells paved the way for immunotherapy [17–19]. Checkpoint inhibitors targeted against the PD-L1/PD-1 axis demonstrated remarkable success in treatment of various cancer types including HNSCC. PD-L1 is a transmembrane protein on the surface of antigen presenting cells and tumor cells, and on the other hand is expressed on the surface of immune-related lymphocytes. Binding of PD-L1 to PD-1 inhibits proliferation and cytotoxicity of lymphocytes [20–22]. To assess the probability of treatment success, the histopathological PD-L1 expression is routinely used to administer checkpoint inhibitor treatment in HNSCC [23]. A meta-analysis of PD-L1 expression and response to checkpoint inhibitor therapy confirmed a better overall survival and tumor response in HNSCC patients with high PD-L1 expression [24]. In SCCUPs checkpoint inhibitors are not routinely administered and the expression of PD-L1 has not been evaluated [25].

Therefore, the aim of this retrospective study was to compare SCCUPs of the head and neck and oropharyngeal carcinomas with regard to the expression of PD-L1 and p16 and the composition of the tumor microenvironment including CD3- and CD8-positive lymphocytes. This could help finding a rationale for targeted therapy of SCCUPs of the head and neck and ultimately improve prognosis of this group of patients.

## Materials and methods

### Patient cohort

In this study a total of 97 patients were included, among them 47 cases with primary oropharyngeal squamous cell carcinomas and 50 cases with lymph node metastases of cancer of unknown primary (CUP). Patients were treated in the Department of Otorhinolaryngology/Head and Neck Surgery, Klinikum rechts der Isar, Technical University of Munich between November 2001 and September 2013. From all patients formalin fixed and paraffin embedded (FFPE) material of resection specimen were obtained from the Institute of Pathology, Technical University Munich, Germany. Clinical and pathological data were gathered in retrospective using patients’ files and electronic records. All patients were followed up regularly in the university hospital. The period of time between initial diagnosis and time of death/last follow-up was used for calculating overall survival. The age of patients in this study ranged from 37.12 to 83.11 years (Median age 60.89 ± 11.9 years, Average age 60.81 ± 9.8 years). The average follow-up period was 4.59 ± 2.6 years (Median 4.76 years ± 2.7) for Overall Survival (OS) and 2.85 years ± 2.8 years (Median 2.03 ± 3.7 years) for Progression free Survival (PFS). Ethical approval was obtained from the ethics committee of the Technical University of Munich (reference number 474/18S).

### Immunohistochemistry of PD-L1, CD3, CD8

FFPE tissue was cut with the microm HM 355 S (International GmbH, Walldorf, Germany) into 2–3 μm thick sections and deparaffinized at 65 °C. Immunohistochemical staining was conducted with a VENTANA BenchMark GX with PD-L1 primary antibody (US Biological, Salem, MA, USA), CD3 primary antibody (1:400, Cell Marque, Rocklin, CA, USA), CD8 primary antibody (1:25, Thermo Fisher Scientific, IL, USA), p16 primary antibody (US Biological, Salem, MA, USA). Slides were counterstained with hematoxylin. After dehydration by immersion in the ethanol series and xylol (2 min each) the slides were examined under light microscopy by two independent researchers. 3 high power fields of tumor in each section were chosen. Tonsil tissue was used as control. Exemplary images of the PD-L1 staining are depicted in Supplementary Fig. 2. The Tumor Proportion Score (TPS) assessed the membranous expression of PD-L1 in tumor cells. Sections with a percentage of expression ≥ 1% were designated as PD-L1 positive, since this threshold was used in the KEYNOTE-012 study of PD-1 inhibition in HNSCC [21]. Tissue with a strong and diffuse nuclear and cytoplasmic p16 staining of more than 70% of tumor cells were considered p16 positive. The area of tumor covered by CD3- and CD8-positive tumor-infiltrating lymphocytes was evaluated in five high-power fields to estimate the impact of the tumor microenvironment on the prognosis of CUP and OPSCC patients. The median number of TILs was used to categorize HNSCC and CUP into high and low infiltration.

To obtain definitive evidence of HPV infection DNA in situ-hybridization (ISH) was applied. The HPV viral types 16, 18, 31, 33, 35, 45, 52, 56, 58, and 66 were detected with the Inform HPV III Family 16 Probe kit (Ventana Medical Systems, AR, USA) according to the manufacturer’s instructions. HPV ISH was interpreted positive when nuclear staining was detected in the infected tumor cells. In addition, the expression of EBV RNA was detected in squamous cell CUPs with the Inform EBER early RNA kit (Ventana Medical Systems, AR, USA) according to the manufacturer’s instructions. A threshold of greater than 30% was considered positive.

### Statistical Analysis

Kaplan–Meier survival analysis and log-rank testing was used to compare survival rates for different patient groups and clinical characteristics. Associations were tested with Fisher`s exact test and Bonferroni correction. At *p* < 0.05 the null hypothesis was rejected, and the result was considered statistically significant. Statistical calculation was performed using Prism 8 (GraphPad Software, La Jolla, CA, USA).

## Results

### Correlation between PD-L1 and pathological data

For the analysis, patients were grouped into PD-L1 positive and negative according to TC score. For the further analysis a threshold of 1% positive stained cells was chosen. The results of staining intensity in percent are depicted in Table 1a. The clinical and pathological characteristics of the cohort are depicted in Table 1b.

**Table 1:** (A) Expression of PD-L1 in cancer cells of CUP and HNSCC patients; (B) depiction of clinicopathological characteristics of the 47 oropharyngeal squamous cell carcinoma and 50 CUP patients included in this study

(A)								
	CUPs				OPSCC			
	Expression of PD-L1				Expression of PD-L1			
	< 1%	1–5%	5–50%	> 50%	< 1%	1–5%	5–50%	> 50%
Abs	16	16	10	8	32	8	7	0
%	32.0%	32.0%	20.0%	16.0%	68.1%	17.0%	14.9%	0%

(B)									
	CUP					OPSCC			
	Overall	PD-L1		*p* value (Fishers exact)		Overall	PD-L1		*p* value (Fishers exact)
		–	+				–	+	
		16	34				16	25	
T1/T2	0	0		0		39	27 (57.4%)	12 (25.5%)	
T3/T4	0	0		0	n.a	8	5 (10.6%)	3 (6.4%)	0.6974
N0	0	0		0		19	14 (29.8%)	5 (10.6%)	
N1–3	0	0		0	n.a	28	18 (38.3%)	10 (21.3%)	0.5423
M0	45	13 (26.0%)		32 (64.0%)		47	32 (68.1%)	15 (31.9%)	
M1	5	3 (6.0%)		2 (4.0%)	> 0.9999	0	0 (0%)	0 (0%)	> 0.9999
p16 +	11	5 (10.0%)		6 (12.0%)		25	13 (27.7%)	12 (25.5%)	
p16−	39	11 (22.0%)		28 (56.0%)	0.2972	22	19 (40.4%)	3 (6.3%)	0.0146
Male	40	13 (26.0%)		27 (54.0%)		37	26 (55.3%	11 (23.4%)	
Female	10	3 (6.0%)		7 (14.0%)	> 0.9999	10	6 (12.8%)	4 (8.5%)	0.7042
Age > Median	25	8 (16.0%)		17 (34.0%)		23	18 (38.3%)	5 (10.6%)	
Age < Median	25	8 (16.0%)		17 (34.0%)	> 0.9999	24	14 (29.8%)	10 (21.3%)	0.2124
HPV ISH +	11	5 (45.5%)		6 (54.5%)		23	11 (44.0%)	12 (48.0%)	
HPV ISH -	0	0 (0.0%)		0 (0.0%)	> 0.9999	2	1 (4.0%)	1 (4.0%)	> 0.9999
EBV–RNA +	2	1 (2.0%)		1 (2.0%)					
EBV–RNA−	48	47 (94.0%)		1 (2.0%)	0.0792				

Using the TC Score with a cutoff of > 1% positive stained tumor cells, 15 of 47 (31.9%) oropharyngeal squamous cell tumor sections were PD-L1 positive. 34 of 50 (68.0%) CUPs were PD-L1 positive. Exemplary images of negative and positive expression are shown in the Supplementary Fig. 1. Association of CUPs demonstrating a higher expression of PD-L1 than OPSCC was tested with Fisher’s exact test and proved to be significant (*p* = 0.0005). The expression of p16 also differentiated CUPs and OPSCC, with 53.2% of OPSCC being p16-positive compared to 28.2% of CUPs (*p* = 0.0018). High risk HPV DNA detection with in situ hybridization revealed a high concordance with p16 IHC staining (*p* = 0.011). An association between HPV–ISH and PD-L1 expression in p16-CUPs was not detected (*p* > 0.9999). p16-negative OPSCC were more likely to be PD-L1 negative (*p* = 0.0146). In only 2 out of 50 (4%) squamous cell CUPs EBV RNA could be detected.

**Fig. 1:**
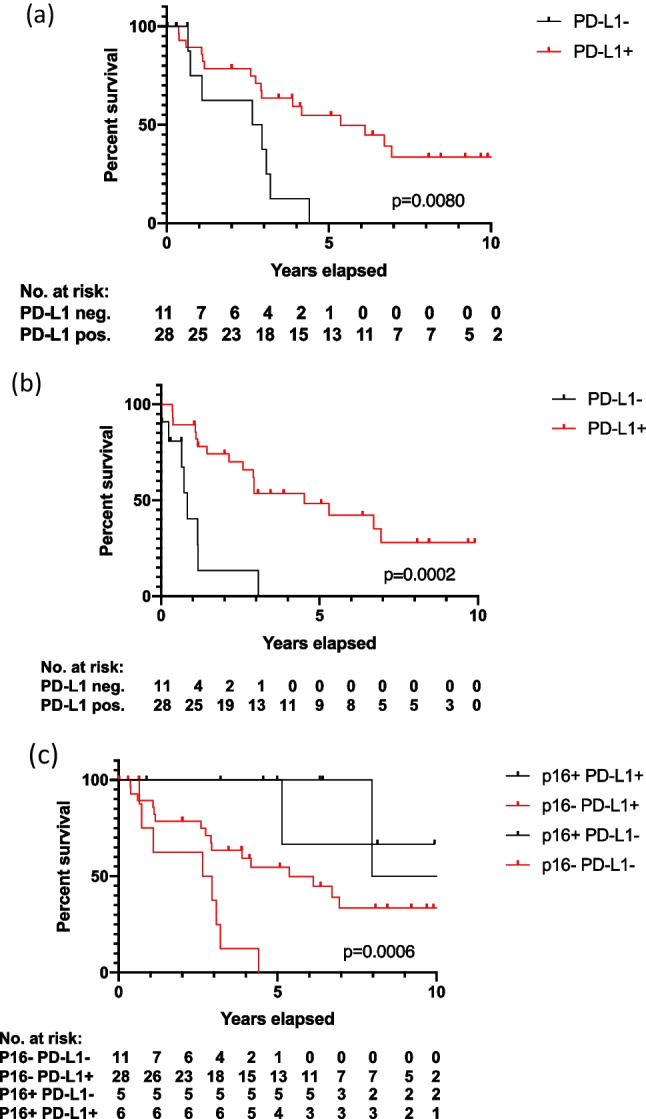
Kaplan–Meier survival analysis of **a** overall survival of p16-negative CUP patients in relation to PD-L1 expression; **b** progression-free survival of p16-negative CUP patients in relation to PD-L1 expression; **c** comparison of Overall Survival of CUP patients stratified by p16 and PD-L1 status

Overall survival (OS) of OPSCC was significantly better than OS of CUPs (*p* = 0.0003) (Supplementary Fig. 1). High expression of PD-L1 in CUP patients was not associated with OS (*p* = 0.3107) or PFS (*p* = 0.2249) (Supplementary Fig. 1). Since the expression of p16 is the most important prognostic factor in HNSCC, the HNSCC and SCCUP cases were stratified into p16-positive and p16-negative SCCUPs. In p16-negative CUPs a high expression of PD-L1 was significantly associated with better OS (*p* = 0.0080) and PFS (*p* = 0.0002) (Fig. 1). There was no significant association between p16-positive CUPs and OS or PFS (*p* = 0.617, *p* = 0.8572) (Supplementary Fig. 1).

The immune cell infiltration of CUPs and OPSCC was measured by evaluating the percentage of positive stained CD3- and CD8-positive tumor infiltrating lymphocytes (TILs) covering the tumor area in 5 high power fields. A high infiltration of CD3-positive lymphocytes was not associated with a significantly better OS (*p* = 0.6806) (Supplementary Fig. 1) in CUP patients. The infiltration of CD8-positive lymphocytes did not differentiate the prognosis of CUP patients significantly (*p* = 0.7834) (Supplementary Fig. 1). The average tumor area infiltrated by CD3-positive TILs was greater in CUP than in OPSCC (6.2% vs 10.4%). This could also be observed with CD8-positive TILs (3.0% vs 6.3%).

## Discussion

In this study, the PD-L1 expression of SCCUPs and OPSCC was compared for the first time. In a second step immune infiltration was assessed to characterize CUPs and get a better picture of underlying immunological mechanisms in this heterogeneous cancer. CUP tissue demonstrated a significantly higher PD-L1 expression (TC) and a stronger infiltration with lymphocytes in this study. This is important, as conventional therapy has high recurrence rates with considerable side effects, whereas the new group of checkpoint inhibitors had remarkable success in treatment of HNSCC and various solid tumors [26–28]. The expression of PD-L1 is the key to drug-induced inhibition of PD-L1, effectively hindering the tumor from inhibiting the immune system. A systematic review and meta-analysis of efficacy and safety in metastatic cancer of solid primaries identified PD-L1 inhibitors as a preferable treatment option. The treatment was even more advantageous in male patients, patients under the age of 65, and current or former smokers [29]. Since the peak incidence of CUP is between 60 and 64 years, in contrast to multiple other cancer entities with higher peak incidences between 80 and 84 years [30], and smoking and alcohol consumption further increases the risk of SCCUP [31], checkpoint therapy could improve the outcome in SCCUP patients. However, a clear rationale for a study focusing on checkpoint therapy in SCCUPs is still missing.

In a former study, PD-L1 expression has been evaluated in the large group of general CUPs (including cases outside of head and neck area) and a positive expression has been detected in up to 28% of CUPs using immunohistochemistry [32]. In that analysis, however, only 30 cases (8% of total cases) represented squamous cell carcinoma. In the mentioned study, a cut off for PD-L1 positivity of ≥ 5% and a different antibody was used. This study analyzes to the knowledge of the authors the largest group of head and neck squamous cell CUPs for PD-L1 expression [32]. Since the expression of PD-L1 is predictive for successful checkpoint inhibitor therapy [33], based on our data, substantial proportion of SCCUP patients could potentially benefit from treatment with checkpoint inhibitors. This is even more relevant for the group of p16-negative patients, which is associated with a worse prognosis [34]. In this study PD-L1 expression differentiated p16-negative patients into a high and low risk group. This finding lays the foundation for a prospective study analyzing PD-L1 expression in head and neck squamous cell CUPs and treatment according to PD-L1 status. For the bigger group of general CUPs, a first study investigating the effect of targeted therapy, including the PD-L1 inhibitor Atezolizumab, has already been started. In this Phase II randomized clinical trial (NCT03498521) unfortunately SCCUP are excluded [16, 35]. This study demonstrates the high expression of PD-L1 in CUP tissue, giving hope that immune checkpoint inhibition could improve survival in at least a relevant proportion of p16-negative squamous cell CUP patients.

To further decipher the tumorigenesis of SCCUP, p16 expression was analyzed. Of all the head and neck malignancies, HPV infection is most common in OPSCC and is, therefore, an important hint toward an oropharyngeal origin of a SCCUP [12, 16]. Some reports showed up to 90% of squamous cell CUPs being p16 positive [36, 37]. This finding could not be replicated by this study, with only 22% of CUPs, and 53% of OPSCC being p16 positive. A potential explanation for this lies in epidemiological trends regarding the percentage of population infected with HPV. In Germany, the number of OPSCC associated with HPV infection increased from 11.5% between 1988 and 2008 to 55.0% between 2004 and 2009 [38, 39]. This finding of high HPV-attributable fractions is important for two main reasons. First, this suggests similarities in the process of carcinogenesis of SCCUPs and OPSCC. In case of SCCUP being a regressed primary with only microscopic remnants, this leads to the oropharynx as the most likely region of origin. Being able to identify the tissue of origin also enables more therapeutic options, including the use of targeted radiation therapy. Radiation of the pharynx and cervical lymph nodes with concurrent chemotherapy led to good regional control in patients with cervical lymph node metastasis from an unknown primary site [40, 41]. Similar to previous studies, the results of this study demonstrated a significantly better survival of patients with HPV positive SCCUPs and OPSCC. While HPV infection is associated with better survival in HNSCC, HPV-negative tumors are more difficult to treat and show a high rate of recurrence [42]. HPV-associated oropharyngeal squamous cell carcinoma are more sensitive to chemo- and radiotherapy and demonstrate a higher recurrence-free and overall-survival [34]. In KEYNOTE-012, immunotherapy with Pembrolizumab led to a response of 32% in HPV-positive, and 18% in HPV-negative recurrent or metastatic HNSCC [21, 43]. HPV-negative HNSCC and SCCUPs until this day remain difficult cancer entities to treat, highlighting the need for novel prognostic tools and therapies.

To characterize SCCUPs in more depth and highlight potential similarities and differences between SCCUP and OPSCC, the composition of the tumor microenvironment was analyzed. Investigating the infiltration of CUPs with TILs revealed a strong infiltration of SCCUPs with immune cells compared to OPSCC. An association of immune cell infiltration with better survival could not be detected. This difference in immunological properties may also contribute toward the rationale of a more targeted therapy with checkpoint inhibitors in the future, since the tumor microenvironment, and more specifically, the infiltration with lymphocytes was associated with the response to checkpoint inhibitor therapy in certain types of cancer [44, 45]. In a recent publication, the therapeutic effect of checkpoint inhibitor therapy could even be predicted by observing the levels of CD8/CD68/CD163/PD-L1 positive cells in non-small cell lung cancer [46]. A prospective study of SCCUP treatment with checkpoint inhibitors should, therefore, take the composition of the tumor microenvironment into account.

## Conclusions

In this study, for the first time, a higher expression of PD-L1 and strong lymphocyte infiltration was observed in SCCUPs compared to OPSCC. These findings support a prospective study evaluating the success of immune therapy in cancer of unknown primary patients of the head and neck.

## Supplementary Information

Below is the link to the electronic supplementary material.

Supplementary file1 (DOC 3286 KB)

## Data Availability

Data available within the article or its supplementary materials.
