# Supplementary material for: Comparison of PD-L1 expression in squamous cell cancer of unknown primary and oropharyngeal squamous cell carcinoma
Source: Eur Arch Otorhinolaryngol. 2022 Dec 28;280(4):1991–7. doi: 10.1007/s00405-022-07775-z (PMC9988788; doi:10.1007/s00405-022-07775-z)
Supplement: Supplementary file 1 — Supplementary file1 (DOC 3286 KB) [file 405_2022_7775_MOESM1_ESM.doc]

| Supplementary Figure 2 |
| --- |
| (A) |
| 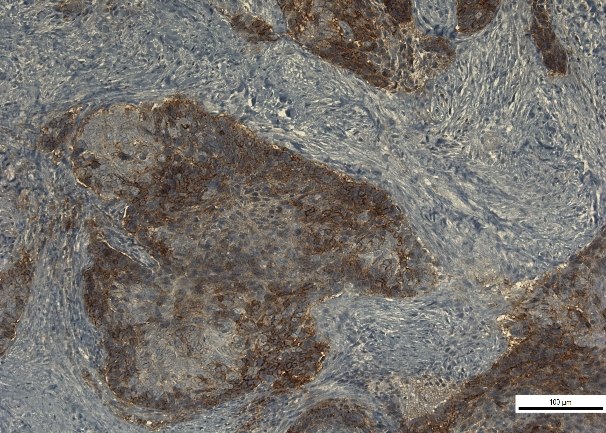 |
| (B) |
| 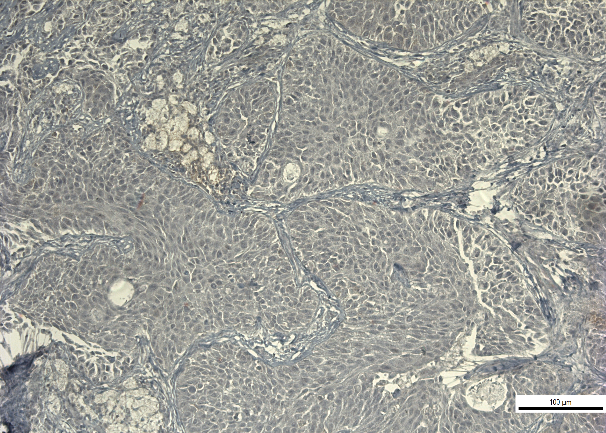 |
| (C) |
| 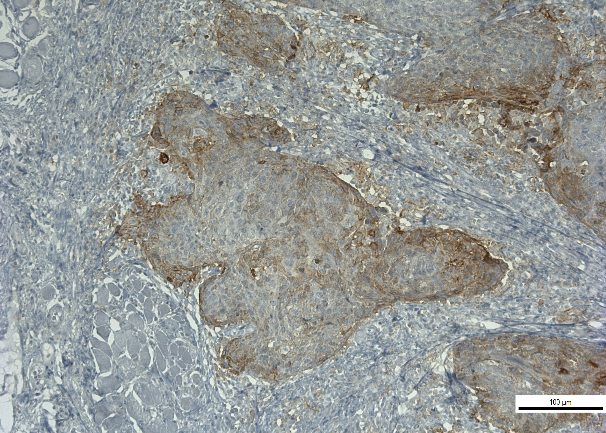 |
| (D) |
| 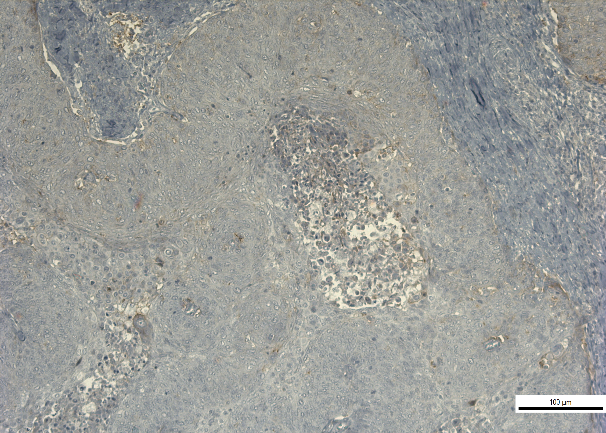 |

**Supp. Fig. 1** Exemplary images of immunohistochemical staining (a) PD-L1 positive CUP; (b) PD-L1 negative CUP; (c) PD-L1 positive OPSCC; (d) PD-L1 negative OPSCC.

| Supplementary Figure 1 |
| --- |
| (A) |
|  |
| (B) |
|  |
| (C) |
|  |
| (D) |
|  |
| (E) |
|  |
| (F) |
|  |
| (G) |
|  |
| (H) |
|  |

**Supp. Fig. 1** Kaplan Meier survival analysis of (A) OS of CUP patients and OPSCC patients; (B) OS of p16-positive CUP patients (C) PFS of p16-positive CUP patients (D) PFS of CUPs and OPSCC patients; (E) OS of CUP patients and PD-L1; (F) PFS of CUP patients and PD-L1; (G) OS CUP patients and CD3 expression; (H) OS CUP patients and CD8 expression.
